# Serum Response Factor Accelerates the High Glucose-Induced Epithelial-to-Mesenchymal Transition (EMT) via Snail Signaling in Human Peritoneal Mesothelial Cells

**DOI:** 10.1371/journal.pone.0108593

**Published:** 2014-10-10

**Authors:** Lijie He, Weijuan Lou, Lihua Ji, Wei Liang, Meilan Zhou, Guoshang Xu, Lijuan Zhao, Chen Huang, Rong Li, Hanmin Wang, Xiangmei Chen, Shiren Sun

**Affiliations:** 1 Department of Nephrology, Xijing Hospital, the Fourth Military Medical University, Xi'an, Shaan xi, China; 2 State Key Laboratory of Cancer Biology, Xijing Hospital, the Fourth Military Medical University, Xi'an, Shaan xi, China; 3 Department of Nephrology, Xingyuan Hospital, the Fourth Hospital of Yulin, Yulin, Shaan xi, China; 4 Department of Nephrology, the Ninth Hospital of Xi'an, Xi'an, Shaan xi, China; 5 Department of Nephrology, State Key Laboratory of Kidney Diseases, Chinese PLA General Hospital and Medical College, Beijing, China; National Centre for Scientific Research “Demokritos”, Greece

## Abstract

**Background:**

Epithelial-to-Mesenchymal Transition (EMT) induced by glucose in human peritoneal mesothelial cells (HPMCs) is a major cause of peritoneal membrane (PM) fibrosis and dysfunction.

**Methods:**

To investigate serum response factor (SRF) impacts on EMT-derived fibrosis in PM, we isolated HPMCs from the effluents of patients with end-stage renal disease (ESRD) to analyze alterations during peritoneal dialysis (PD) and observe the response of PM to SRF in a rat model.

**Results:**

Our results demonstrated the activation and translocation of SRF into the nuclei of HPMCs under extensive periods of PD. Accordingly, HPMCs lost their epithelial morphology with a decrease in E-cadherin expression and an increase in α-smooth muscle actin (α-SMA) expression, implying a transition in phenotype. PD with 4.25% glucose solution significantly induced SRF up-regulation and increased peritoneal thickness. In immortal HPMCs, high glucose (HG, 60 mmol/L) stimulated SRF overexpression in transformed fibroblastic HPMCs. SRF-siRNA preserved HPMC morphology, while transfection of SRF plasmid into HPMCs caused the opposite effects. Evidence from electrophoretic mobility shift, chromatin immunoprecipitation and reporter assays further supported that SRF transcriptionally regulated Snail, a potent inducer of EMT, by directly binding to its promoter.

**Conclusions:**

Our data suggested that activation of SRF/Snail pathway might contribute to the progressive PM fibrosis during PD.

## Introduction

Peritoneal dialysis (PD) is currently used as a chronic, life-sustaining treatment by approximately 197,000 end-stage renal disease (ESRD) patients, or 11% of the global dialysis population [1]. Long term PD is limited because of the structural and functional changes in the peritoneal membrane (PM) induced by PD fluids (PDFs), which contain high concentrations of glucose that finally lead to a loss of ultrafiltration [2]. The epithelial-to-mesenchymal transition (EMT) is a complex, step-wise phenomenon beneficial for normal wound healing [3] but detrimental in fibrogenic diseases [4], such as peritoneal fibrosis. Biomarkers for EMT have been identified and categorized, including the loss of the epithelial adhesion protein E-cadherin and upregulation of the mesenchymal marker α-smooth muscle actin (α-SMA) [5]. Preventing EMT could ameliorate peritoneal fibrosis, preserving the PM during PD [6].

Serum response factor (SRF), a member of a conserved DNA-binding protein family, is a master switch for the expression of contractile and cytoskeletal genes in virtually all cells across diverse species [7]. SRF plays important roles in diverse pathological processes, including EMT-derived tumor metastasis and fibrosis. For example, SRF expression in hepatocellular carcinoma (HCC) cells that can undergo EMT may play an enhanced role in tumor progression [8]. Overexpression of SRF in colorectal carcinoma cells is associated with the modulation of E-cadherin/β-catenin expression and may enhance metastasis [9]. In addition, SRF translocation into nuclei may contribute to myofibroblast differentiation in human lung fibroblasts and cardiac fibrosis [10]–[12]. SRF targets, which contain a serum response element (SRE), are activated when SRF is in the nucleus [13], [14]. CCG-1423 is a specific inhibitor of Rho pathway-mediated signaling and activation of SRF transcription. CCG-1423 selectively inhibits DNA synthesis, proliferation and invasion of Rho-overexpressing cell lines. Recently, the SRF inhibitor (CCG-1423) was suggested to be a promising compound as a novel pharmacological tool in preventing prostate cancer progression [15].

The high glucose (HG)-induced EMT of HPMCs acts as a key process in peritoneal membrane fibrosis and dysfunction. Mediated by factors including E-cadherin, α-SMA and Snail, epithelial cells may lose their epithelial characteristics and gain mesenchymal cell properties in response to certain stimuli [16], [17]. However, to date, whether SRF is involved in EMT-mediated PM deterioration remains incompletely understood. Here, we will firstly demonstrate the role and mechanism of SRF in the EMT-derived peritoneal fibrosis.

## Methods

### Ethics Statement

The study protocol conformed to the ethical guidelines of the 1975 Declaration of Helsinki and was approved by the ethics committee of Xijing Hospital. Written informed consent was obtained from each patient. The Ethics Committee for Animal Experiments of the Fourth Military Medical University approved all animal work (Permit number: 20120023) and the experimental protocols strictly complied with the institutional guidelines and the criteria outlined in the “Guide for Care and Use of Laboratory Animals”. And all efforts were made to minimize the number of animals used and their suffering, in accordance with the ethical guidelines for animal research. All surgery was performed under sodium pentobarbital anesthesia.

### HPMC isolation and culture

Human mesothelial cells obtained from the effluents of patients undergoing CAPD in our PD center in the Department of Nephrology, Xijing Hospital were collected and cultured using a previously described method [18]–[20]. The use of human peritoneal fluid in this study was approved by the institutional review board of the Fourth Military Medical University and was done in accordance with international guidelines for the use of human tissues. The inclusion criteria were age less than 65 years, duration of CAPD longer than 3 months, no peritonitis in the last 6 months, the use of 1.5% glucose dialysis solution, and no history of abdominal surgery or long-term use of an immunosuppressant (Table S1). The CAPD patients were sorted into three groups according to the duration of dialysis (Group 1 was 3-6 months, Group 2 was 7–24 months, and Group 3 was longer than 25 months).

The immortal human HPMC line (ATCC, Rockville, MD), which is an untransformed HPMC line that yields data similar to the results obtained using primary cells, was cultured in Earle's M199 medium. EMT was induced in immortal HPMCs with HG (0.5 ng/mL) (R&D Systems, Minneapolis, MN), as described previously [21]–[23].

### Peritoneal exposure model in rats and immunofluorescence analyses of the peritoneum

A chronic infusion model of non-uremic animal PD was used, as described previously [23]. Briefly, 24 male Sprague-Dawley rats (250 to 300 g) were divided into three groups with eight rats each. The control group was injected with normal saline (100 ml/kg/d) each day for 6 weeks; the HG-PDF group was injected with 4.25% glucose dialysis solution (100 ml/kg/d); and the SRF inhibitor group was injected with 4.25% glucose dialysis solution and CCG-1423 (0.03 mg/kg/d). Lipopolysaccharide (LPS, Sigma, final concentration of 5 µg/L) was used at 8, 9, and 10 days in the HG-PDF and SRF inhibitor groups [6], [23]. The SRF inhibitor was purchased from Calbiochem (EMD, Darmstad, Germany). The parietal peritoneum of the abdominal wall was fixed and embedded in paraffin as described in the Concise Methods section. For immunofluorescence microscopy, 3 µm tissue sections were incubated with primary antibodies against E-cadherin, α-SMA or SRF. Rats were manipulated and cared for according to NIH Animal Care and Use Committee guidelines in the Experiment Animal Center of the Fourth Military Medical University (Xi'an, Shanxi, Province, P.R. China).

### Western blotting

Total protein extracts from cultured cells were loaded on SDS-PAGE gels, and Western blotting was performed according to a standard protocol. The antibodies used were as follows: anti-p-SRF (Cell Signaling Technology, Inc) diluted 1∶500; anti-SRF diluted 1∶80; anti-E-cadherin, anti-α-SMA, and anti-Snail diluted 1∶50 (Santa Cruz Biotechnology); and anti-β-actin (Sigma) diluted 1∶4000. The antibody staining was performed as described previously [23], [24].

### Confocal microscopy and immunofluorescence

Cultured cells were grown on 0.8 cm×0.8 cm slides, rinsed with ice-cold phosphate-buffered saline and fixed with 4% paraformaldehyde. Immunofluorescence staining was performed on paraffin-embedded sections or cultured cells, as described previously [24]. Samples were immunostained with anti-SRF, anti-α-SMA, anti-E-cadherin or anti-Snail antibodies (Santa Cruz Biotechnology, CA) and then counterstained with FITC-labeled IgG secondary antibodies (Invitrogen). The nuclei were visualized using DAPI. Images were obtained using an Olympus microscope and IM50 imaging software (Leica) or using an FCS confocal laser scanning microscope (LSM510; Carl Zeiss MicroImaging). All sections or slide were examined and scored independently by two investigators without knowledge of the outcome.

### Plasmid construction and cell transfection

The siRNA plasmids that recognize human SRF (sc-36563) were purchased from Santa Cruz Biotechnology for the transient transfections. The full-length human SRF plasmids were a gift from Dr. Eric Olson (Department of Molecular Biology, UT Southwestern Medical Center at Dallas, USA). The transfections were performed using Lipofectamine 2000 (Invitrogen, Carlsbad, CA), according to the manufacturer's instructions. Real-time PCR or Western blot analysis was used to assess the transfection efficiency.

### Chromatin immunoprecipitation and quantitative PCR

ChIP was performed as previously described [25], [26]. Chromatin fragments were co-immunoprecipitated from HG-treated HPMCs and control lysates with the appropriate amount of SRF antibody or an equivalent amount of rabbit IgG (8 µg) as a control. The purified DNA fragments were used as templates for PCR amplification. Real-time PCR was performed using an ABI 7900 Sequence Detection System (Applied Biosystems) and the SYBR Green PCR core reagent kit (Applied Biosystems). The specific primers were as follows: Snail (SRE1) ChIPF1, 5′-CGTCTGTCTCCCTCACTGGACC-3′, Snail (SRE1) ChIPR1, 5′-CTCTCGGCGGCTTGAAATGC-3′, Snail (SRE2) ChIP F2, 5′-GCCCGGGCTCTCACCGCCAC-3′, and Snail (SRE2) ChIP R2, 5′-GCAGCAGCGCCGCCAACTCC-3′. The values were normalized to the input and expressed as a fold increase relative to the IgG control.

### DNA sequencing and plasmid construction for the promoter analysis

The snail promoter region from −1 to −1000 was cloned into the pGL3 luciferase vector (Promega) (Promega, Madison, WI, USA). The primers used to create the mutant reporters (MTSNAIL-1, MTSNAIL-2, MTSNAIL-3) are as follows: mutSnailF1: 5′-CGTGCTGGGCGCTCCGTAAACAATCGATCGATCGAGGAACGGGTGCTCTTGGCTAGC-3′, mutSnailR1: 5′-GCTAGCCAAGAGCACCCGTTCCTCGATCGATCGATTGTTTACGGAGCGCCCAGCACG-3′, mutSnailF2: 5′-GCGCGGAGGTGACAAAGGGGCGATCGATCGATCGCCCCGCCCCTCCCACCCCCCACCA-3′, and mutSnailR2: 5′-TGGTGGGGGGTGGGAGGGGCGGGGCGATCGATCGATCGCCCCTTTGTCACCTCCGCGC-3′.

### Transient transfection luciferase assay

For the transient transfection assays, HG-treated HPMCs or control cells were seeded 48 h before transfection. Then, pGL3-basic was transfected with the PRLsv40 plasmid into cells in the presence of Lipofectamine. The luciferase activity in cell lysates collected 48 h after transfection was measured as relative light units with the Fluoroscan Ascent FL (Labsystems, Franklin, MA, USA) using the Dual-Luciferase Assay System (Promega). The relative luciferase activity was defined as the ratio of the firefly luciferase activity/mean Renilla luciferase activity for each construct relative to the pGL3 control vector (Promega) [24].

### Electrophoretic mobility shift assay

Nuclear proteins were isolated using the Nuclear Protein Isolation kit (Panomics). The binding reaction for the electrophoretic mobility shift assay (EMSA) contained 10 mmol/L HEPES (pH 7.5), 80 mmol/L KCl, 1 mmol/L EDTA, 1 mmol/L EGTA, 6% glycerol, 0.5 µg of polydeoxyinosinic-deoxycytidylic acid, 0.5 µg of sonicated salmon sperm DNA, 2 pmol of a double-stranded oligonucleotide of AR/GR HRE conjugated to IR fluorophore IRDye 700 (LI-COR), and 10 µg of the nuclear extract. To verify the specificity of the AR (GR) protein-DNA complexes, a binding reaction was performed using increasing concentrations of non-conjugated HRE (cold probe) and alternating with a mutated HRE conjugated to IRDye 700. A DNA-binding reaction was performed at 16°C for 30 min in a final volume of 20 µL. The DNA-protein complexes were analyzed by Tris-borate EDTA electrophoresis on 6% polyacrylamide gels containing 0.5% glycerol. The DNA-protein complexes were visualized using an Odyssey IR scanner (LI-COR) with a 680-nm scanner channel, and linear graphs were constructed with the LI-COR Odyssey software for quantitative image analysis (LI-COR) [24].

### Reverse-transcription PCR

Total RNA was extracted using the RNeasy kit (Qiagen GmbH, Hilden, Germany), and cDNA was obtained from 500 ng of total RNA using an Omniscript RT kit (Qiagen). Quantitative PCR was performed using a LightCycler (Roche Diagnostics GmbH, Mannheim, Germany), SYBR Green kit (Roche Diagnostics GmbH) and the following specific primer sets: GAPDH, F: 5′-CCTCAAGATCATCAGCAAT-3′ and R: 5′-CCATCCACAGTCTTCTGGGT-3′ (used for normalization); SRF, F: 5′-AAACTGCAGCCCATGATCACC-3′ and R: 5′-CTTCAAAGCCAGTGGCACTCA-3′; CDH1, F: 5′-TTCCCTCGACACCCGATTC-3′ and R: 5′-TAGGTGGAGTCCCAGGCGTA-3′; α-SMA, F: 5′-AAGATGACCCAGATCATGTT-3′ and R: 5′-TCAT- AGATGGGGACATTGT-3′; and Snail, F: 5′-ACCCCAATCGGAAGCCTAACT-3′ and R: 5′-GGTCGTAGGGCTGCTGGAA-3′. Experiments were performed in duplicate. After amplification, the PCR products were confirmed by melting curve analysis and gel electrophoresis.

### Statistical analysis

The bands from Western blotting or real-time PCR were quantified using Quantity One software (Bio-Rad, Hercules, CA, USA). The numerical data are presented as the mean ± SE using SPSS 12.0 software (Chicago, IL, USA). Differences between the means were assessed using Student's t-test, an ANOVA or the χ^2^ test. Significant differences were determined using an analysis of variance followed by a post-hoc comparisons with Fisher's protected least-significant-difference test. The data were significant different if P<0.05.

## Results

### 1. Nuclear translocation of SRF occurs in human peritoneal mesothelial cells and morphological and biomarker alterations are consistent with EMT in continuous ambulatory peritoneal dialysis (CAPD) patients

**Figure 1:**
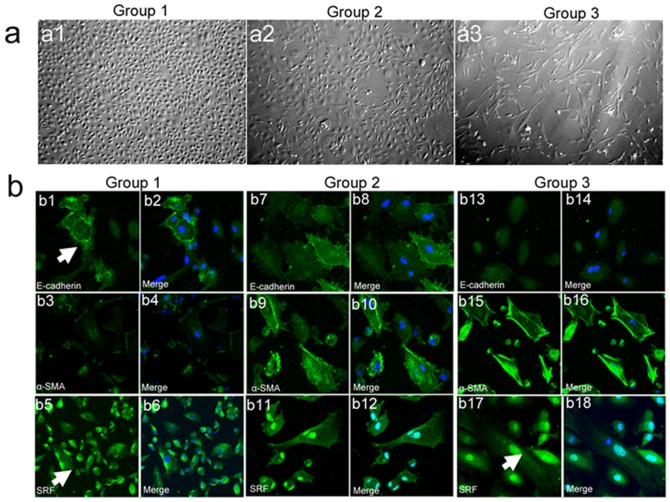
SRF expression in PD effluent–derived HPMCs. (a) Phase contrast microscopy shows different morphological characteristics of HPMCs. (a1) Effluent-derived HPMCs from Group 1 patients with a cobblestone-like morphology. (a2) Effluent-derived HPMCs from Group 2 patients with mixed transitional morphology. (a3) Effluent-derived HPMCs from Group 3 patients with a fibroblast-like morphology. Magnification is 200×. (b) Differences in the EMT markers E-cadherin, α-SMA and SRF expression in HPMCs with different phenotypes from the three groups. Magnification is 200×.

Morphological features of human peritoneal mesothelial cells were markedly variable, ranging from a cobblestone-like appearance to mixed cell populations and fibroblast-like cells (Table 1, Figure 1a). Of the 42 effluent cultures evaluated, 76.47% (13/17) of Group 1 had a cobblestone shape, 70.00% (7/10) of Group 2 showed a mixed morphology (cobblestone and fibroblast-like morphology), and 86.67% (13/15) of Group 3 were fibroblast-like cells. To verify the phenotype of the HPMCs from the PD effluents, we examined expression of E-cadherin, a typical epithelial marker, and α-SMA, a typical mesothelial marker, by immunofluorescence. Effluent cells from Group 1 with a cobblestone-like appearance had higher E-cadherin expression and lower α-SMA expression. In contrast, fibroblast-like cells from Group 3 showed the highest α-SMA expression without E-cadherin expression. The effluent cells from Group 2 patients were in between with a mixed appearance. SRF was primarily localized within the cytoplasm of the HPMCs from Group 1 (70.59%, 12/17). However, SRF translocated to the nuclei of cells from Groups 2 and 3 (Table 1, Figure 1b). These morphological changes, along with the E-cadherin downregulation and α-SMA upregulation in the mesothelial cells derived from the effluent, implied potential EMT after extensive PD.

**Table 1:** Expression and location of SRF in primary HPMCs.

Characteristic	Group 1 (n = 17)	Group 2 (n = 10)	Group 3 (n = 15)	P
CAPD times	3–6 m	6–24 m	>24 m	
Morphology of primary HPMCs				0.000
Cobblestone morphology	13	1		
Mixed morphology	4	7	2	
Fibroblast-like morphology		2	13	
Location of SRF in primary HPMCs				0.000
Located in cytoplasm	12	2	0	
Located both in cytoplasm and nuclei	4	6	4	
Located in nuclei	1	2	11	

### 2. Benefits of SRF inhibition in sustaining PM function in rats undergoing PD

**Figure 2:**
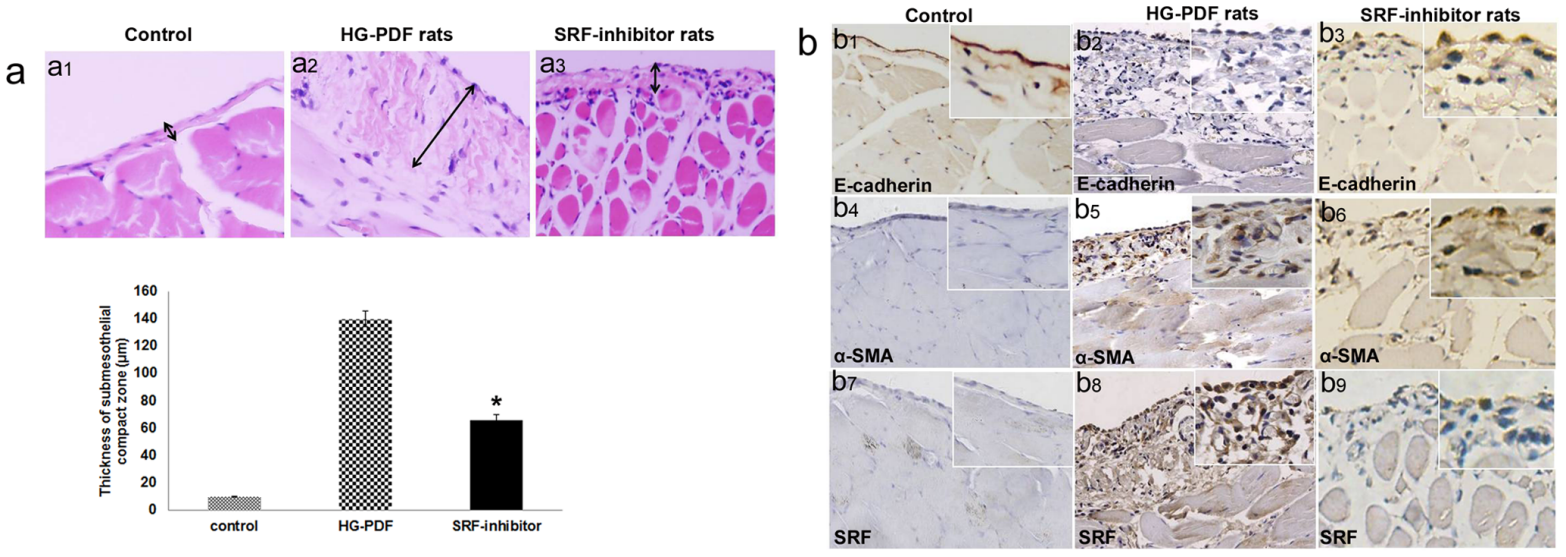
Inhibition of SRF by CCG-1423 ameliorates PD-induced PM fibrosis *in vivo*. (a) Rats received a daily instillation of PD fluid or saline for 6 weeks. After that period, samples were prepared and analyzed as described in the Concise Methods section. (a1) As measured by HE staining, saline exposure resulted in little change in PM. (a2) Exposure to 4.25% PD fluid resulted in an increase in the thickness of the PM. (a3). CCG-1423 treatment significantly reduced HG-PD effects. The histogram showed the thickness for HE staining in the submesothelial compact zone (mean±SE, n = 8). Magnification is 200×. (b) Staining of the EMT markers E-cadherin, α-SMA and SRF in peritoneal samples by immunohistochemistry reveals that PD fluid exposure induces the EMT process, while CCG-1423 treatment significantly ameliorated EMT-derived fibrosis. Magnification is 200×.

To examine the effect of SRF inhibition in PD, we performed PD in rats with HG-PDF (4.25% glucose dialysis solution) with or without CCG-1423 for 6 weeks. By histopathological examination, the thickness of the submesothelial compact zone in the HG-PDF group (139.9±5.5 µm) was significantly greater than that in the control group (9.7±0.5 µm), significantly less in the SRF-inhibitor group (65.9.7±4.4 µm, P<0.01 versus the HG group). So a loss of the mesothelial cell monolayer with a thicker PM was found in the parietal peritoneum from the untreated PD group compared to the saline control (Figure 2a1, a2), while CCG-1423 significantly preserved peritoneal thickness and mesothelium morphology in the treated group (Figure 2a3, Figure S2).

**Figure 3:**
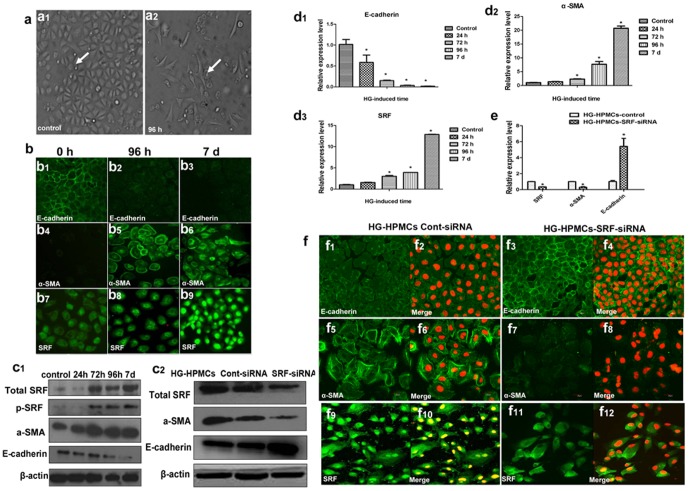
HPMC EMT induced by HG *in vitro* results in increased SRF expression, while knockdown of SRF by SRF-siRNA reverses HPMC EMT *in vitro*. (a) Morphological changes in HPMCs induced by HG stimulation for 96 h compared to the control. Magnification is 200×. (b) Fluorescence microscopy showed the altered location and expression of SRF and EMT markers E-cadherin and α-SMA in HPMCs induced by 0 h, 96 h and 7 d of treatment. Magnification is 200×. (c1) Western blot analysis showing the induction of SRF, p-SRF and EMT marker proteins E-cadherin and α-SMA expression by HG at 0 h, 24 h, 48 h, 72 h, 96 h, and 7 d in immortal HPMCs. (c2) Western blot analysis of SRF in HPMCs which were exposed with HG for 96 h and then transfected with SRF-siRNA or control vector. (d1-3) Induction of E-cadherin, α-SMA and SRF mRNA expression at 0 h, 24 h, 48 h, 72 h, 96 h, and 7 d in immortal HPMCs. Bars in A represent the fold induction over untreated cells and are depicted as the mean +/− S.E. of three independent experiments conducted in duplicate(*P<0.05 vs. control HPMCs). (e) Real-time PCR showing mRNA of SRF, E-cadherin and α-SMA in HPMCs transfected with SRF-siRNA or control vector (*P<0.05 vs. HG-HPMCs-control). (f) Fluorescence microscopy showed the location and expression of the EMT markers E-cadherin, α-SMA and SRF in HG-induced SRF-siRNA-treated HPMCs and control cells. Magnification, 200×.

EMT markers and fibronectin (FN) analysis by immunohistochemistry indicated that HG-PDF downregulated E-cadherin and upregulated α-SMA levels, which were inhibited by the administration of CCG-1423, suggesting EMT was tempered (Figure 2b1-b6, Figure S3). A correlation between PM thickness and SRF expression is shown in Figure 3b7-b9 and 3c. In addition, CCG-1423 significantly attenuated the HG-PDF-induced SRF expression (Figure 2b7–b9).

### 3. Small interfering SRF RNA demoted EMT, while transfection of SRF promoted EMT in cultured HPMCs

To pursue direct evidence of SRF on HG-induced EMT, we manipulated SRF gene expression in cultured immortal HPMCs. When the control HPMCs were incubated with HG medium (60 mmol/L) for 72 h, the cells lost intercellular junctions, scattered, and transformed into a spindled fibroblastic shape (Figure 3a, Figure S1). Concordant with our ex vivo and in vivo results, HPMCs lost E-cadherin, gained α-SMA, translocated SRF to nuclei (Figure 3b), and increased SRF at both protein (Figure 3c1) and mRNA levels (Figure 3d1, d2, d3) in response to HG stimulation.

**Figure 4:**
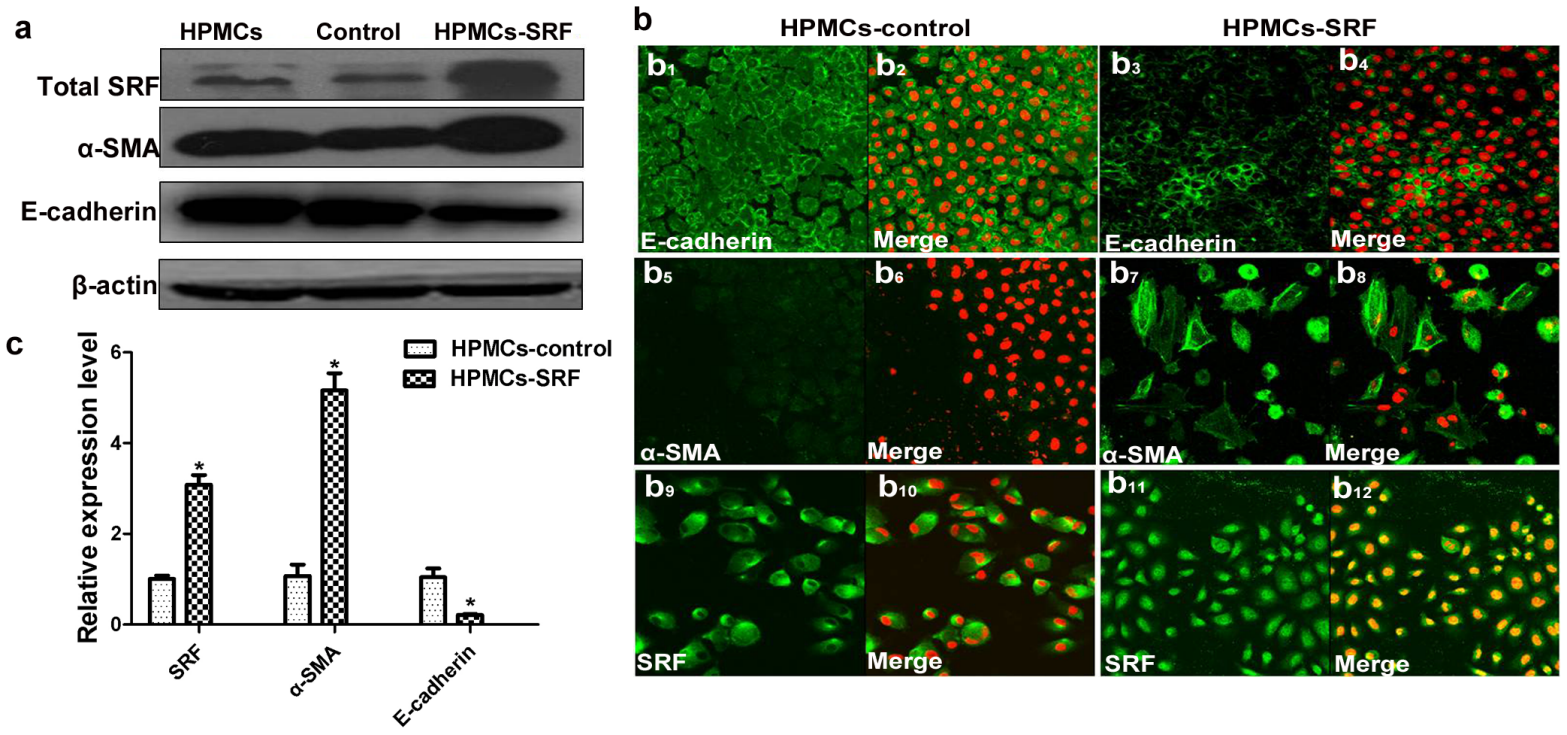
Overexpression of SRF by a SRF plasmid enhances HPMC EMT *in vitro*. (a) Western blot analysis of SRF in HPMCs transfected with a SRF plasmid or control plasmid. (b) Fluorescence microscopy showed the location and expression of the EMT markers E-cadherin, α-SMA and SRF in HPMCs transfected with a SRF plasmid or control plasmid. Magnification is 100×. (c) Real-time PCR showing mRNA of SRF, E-cadherin and α-SMA in HPMCs transfected with a SRF plasmid or control plasmid (*P<0.05 vs. control).

Small interfering SRF RNA (SRF-siRNA) in HPMCs prevented all of the above HG-induced alterations (Figure 3c2, e, f). In contrast, over-expression of SRF by transfecting the full-length human SRF plasmid [26] into immortal HPMCs led to significant downregulation of E-cadherin and upregulation of α-SMA protein levels (Figure 4a, 4b), as well as mRNA levels (Figure 4c), which is consistent with the results from HG-induced primary cultured HPMCs. Therefore, the mediation of SRF on EMT in peritoneal mesothelial cells was further supported.

### 4. Snail is a downstream target of SRF in EMT-mediated PM fibrosis

**Figure 5:**
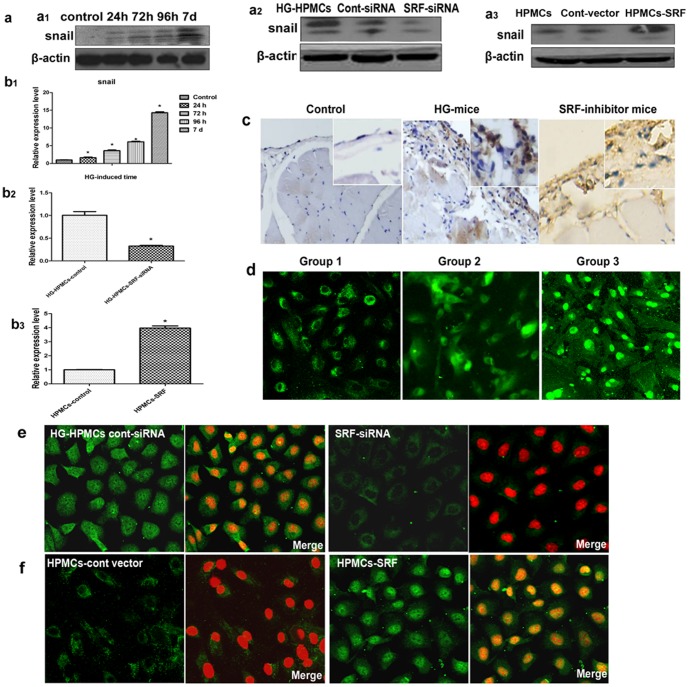
SRF regulates the expression of Snail in HG-induced HPMCs. (a1) Western blot analysis of expression of Snail in HG-induced HPMCs in vitro compared to control HPMCs at 0 h, 24 h, 48 h, 72 h, 96 h, and 7 d. (a2) Western blot analysis of Snail in HPMCs transfected with SRF-siRNA or control vector. (a3) Western blot analysis of Snail in HPMCs transfected with a SRF plasmid or control plasmid. (b1) Real-time PCR showing the expression of Snail in HG-induced HPMCs at 0 h, 24 h, 48 h, 72 h, 96 h, and 7 d. (b2) Real-time PCR showing the expression level of Snail in HG-induced SRF-siRNA-treated HPMCs compared to HG-induced HPMCs. (b3) Real-time PCR showing the expression level of Snail in HPMCs infected with SRF plasmid compared with control HPMCs (*P<0.05 vs. control). (c) The results from immunohistochemistry showed Snail staining in the peritoneal samples of normal rats treated with HG or HG+SRF inhibitor (CCG-1423). Magnification is 200×. (d) Location and expression of Snail in ex vivo HPMCs from patients with different phenotypes from Groups 1, 2 or 3. (e) Fluorescence microscopy showed the location and expression of Snail in HG-induced in vitro HPMCs transfected with SRF-siRNA vector compared with the control vector. Magnification is 200×. (f) Fluorescence microscopy showed the location and expression of Snail in HPMCs transfected with a SRF plasmid or the control plasmid in vitro. Magnification is 200×.

To explore the downstream events of SRF-mediated EMT, we assessed target gene sequences for SRF. We found that all possible SRF target genes have one or more highly conserved CArG elements, leading to select Snail as a candidate of the EMT marker in the promoter. Indeed, Snail expression was augmented in HG-induced HPMCs or HPMCs with SRF overexpression both at the protein and mRNA levels, while levels were diminished by siRNA (Figure 5a, b). These data showed that Snail expression might be correlated with SRF expression at the mRNA and protein levels in HPMCs. This upregulation was also found in the Group 3 PD patients compared with Group 1.

In addition, immunohistochemical studies also revealed pronounced Snail staining in the submesothelial layer of the abdominal walls of the PDF rats, which could be attenuated by treatment with the SRF inhibitor CCG-1423 (Figure 5c). Snail translocated from the cytoplasm into the nuclei in ex vivo HPMCs with prolonged PD (Figure 5d). Translocation of Snail expression was recognized in HG-induced HPMCs, SRF-infected HPMCs, and SRF-siRNA-treated HPMC-HG cells in vitro (Figure 5e, f). These findings suggested that Snail could be the downstream target and mediator of SRF in rat peritoneum when exposed to effluent HG-PD.

### 5. SRF transcriptionally regulated Snail expression by directly binding the SNAIL promoter

**Figure 6:**
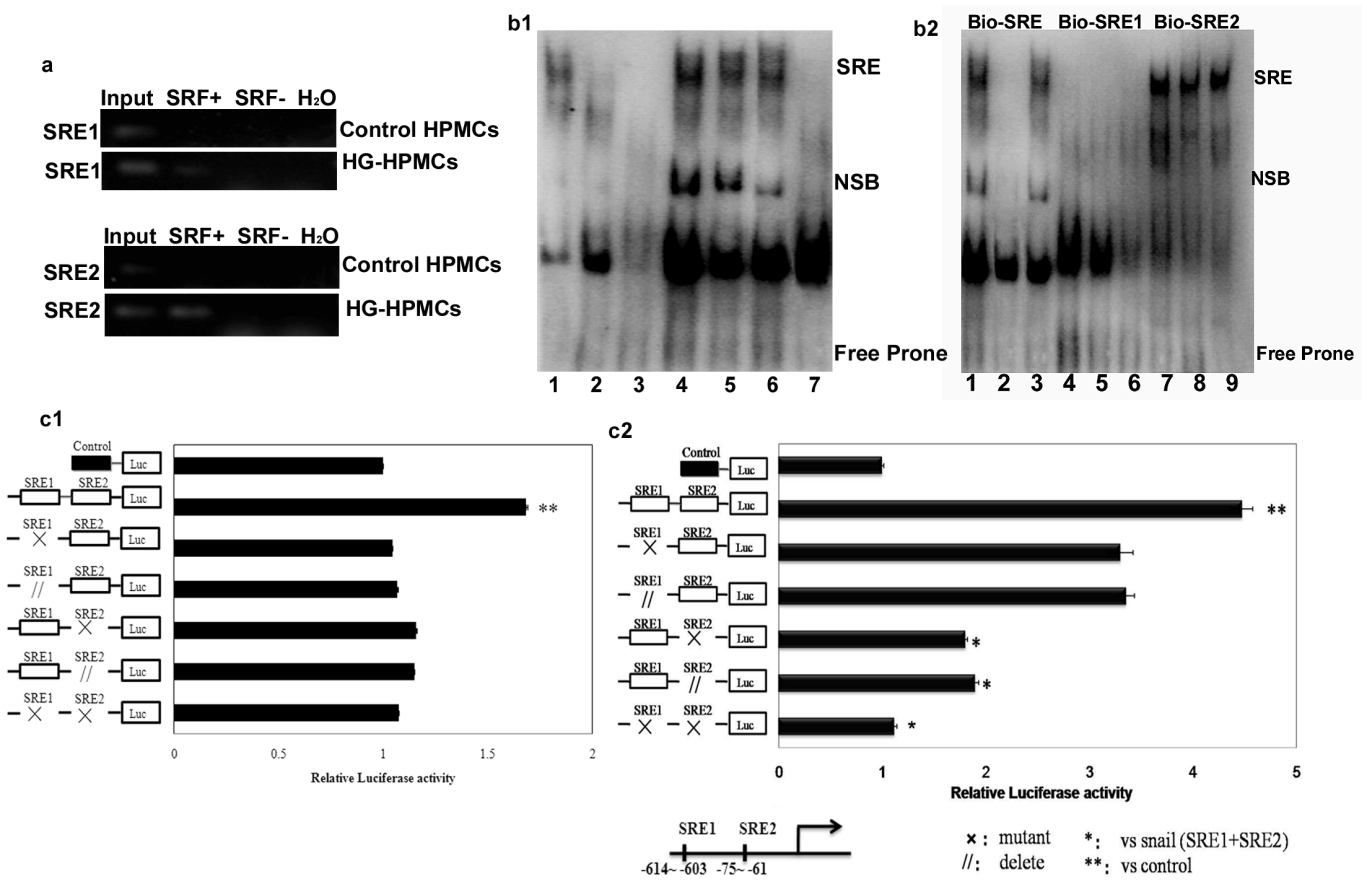
SRF directly induced Snail expression and transcriptional activation in HPMCs. (a) Identification of SRE1 and SRE2 in the SNAIL promoter. Chromatin immunoprecipitation was used to examine SRF binding to the SNAIL promoter in HG-induced HPMCs and the control. Reaction controls included immunoprecipitations performed using a nonspecific IgG monoclonal antibody; PCR was performed using whole cell genomic DNA (Input). A representative example of three independent experiments is shown. (b) EMSA was used to analyze the SRF-binding site induced by HG. (b1) Samples 1 to 7 were induced by HG for 0 h, 5 h, 18 h, 72 h, and 96 h and compared with a positive control and negative control. SRE was bound after inducing for 72 h. (b2) Analysis of the SRF-binding site in the SNAIL promoter by EMSA. Biotin-labeled SRE1 and SRE2 oligonucleotides were used as a probe. Of the two examined putative SRE-binding sites within the SNAIL gene promoter, only SRE2 displayed specific SRF binding in HPMCs induced by HG. Samples 1 to 7 were 72 h+SRE, 72h+SRE + cold-SRE, 72h+SRE + cold SER Mut, 72h+SRE1, 72 h+SRE1+ cold-SRE1, 72h+SRE1+ cold-SRE1 Mut, 72h+SER2, 72h+SER2 +cold-SRE2, and 72h+SER2 +cold-SRE2 Mut. Cold probe, unlabeled SRE; SRE mut, mutated SRE. (c) For the competition assays with SRE1 and SRE2, 200-fold molar excess of the Snail plasmid and mutant SRE1, deleted SRE1, mutant SRE2, deleted SRE2 and mutant SRE1 and SRE2 were used in HPMCs (c1) and HG-induced HPMCs (c2). All results shown are representative of at least three independent experiments. * P<0.05 vs. Snail (SRE1+SRE2) plasmid; ** P<0.05 vs. control plasmid.

In hunting for the molecular basis of the regulation of Snail by SRF, we performed chromatin immunoprecipitation (ChIP) to examine the binding of SRF to CArG elements in Snail in HPMCs. Two serum response elements, SRE1 and SRE2, in the promoter regions of the Snail gene were predicted as candidate binding sites of SRF. Fifty-fold enrichment was set as a threshold for positive SRF binding. Our results showed positive SRF binding at SRE2 in HPMCs. Moreover, the binding signal greatly increased in the HG-induced HPMCs, in agreement with their higher SRF expression (Figure 6a). Electrophoretic mobility shift assays (EMSA) further confirmed that SRF was active and bound to an intronic CArG element after HG incubation for 72 or 96 h (Figure 6b), compared with fewer SRF-containing complexes from the HPMCs in normal medium.

Up-regulated Snail by SRF was further confirmed by luciferase activity assays. In the control HPMCs, transfected full-length SRE (SRE1+SRE2) plasmids could stimulate their luciferase activity from the basal level either in normal medium (P = 0.030) or high glucose medium (P = 0.007) compared to control vectors. Mutation of SRE1 moderately affected their response but without statistical significance (P = 0.061). However, mutation of SRE2 alone or a double mutation of both SRE1+SRE2 eliminated the luciferase activity response to HG (P = 0.013 and P = 0.001 vs. SRE1+SRE2, respectively) (Figure 6c1, c2). These results suggested that the activation of the SRF-Snail signaling occurs via the binding of SRF to Snail predominately at the SRE2 site in its promoter region.

## Discussion

Our results demonstrate that HPMCs from ex vivo effluents or from PD rats in vivo undergo EMT during PD. We determined that SRF promotes EMT-mediated fibrosis and acts as a critical transcription factor for HPMCs. Moreover, infection with SRF-siRNA vectors demonstrated the HG-induced upregulation of E-cadherin and downregulation of α-SMA during EMT in HPMCs in vitro. Furthermore, we predicted and demonstrated that Snail is regulated by SRF, which can directly promote Snail transcription via SRE2. Thus, a novel SRF/Snail pathway promotes EMT-mediated peritoneal fibrosis and acts as a critical transcription factor during HPMC EMT.

Increasing evidence shows that EMT, which was originally thought to be limited to tumor development and metastasis, occurs in other diseases, including kidney, liver and lung fibrosis [27]–[29]. Recently, HPMCs EMT in the peritoneum and effluents of patients undergoing PD was associated with the recurrent use of HG and has been linked to a decline in peritoneal function due to peritoneal fibrosis [4], [30]. Using HG, we sought to reproduce the damage caused by the PD effluent and the profibrotic stimuli induced during peritoneal EMT in vivo and in vitro. HG in the PD effluent may induce EMT in HPMCs and has additional effects on cell morphology [30]. Our results revealed that HG could change HPMC morphology and EMT markers, which as an available model for peritoneal EMT in vitro. We could see that HPMCs lost their epithelial morphology with a decrease in E-cadherin expression and an increase in α-SMA expression, implying a transition in phenotype. Several transcription factors, such as twist, were proved to induce EMT in our previous work [23]. In our study, we found that HG treatment induced SRF expression and increased α-SMA but decreased E-cadherin expression. The activation and translocation of SRF into the nuclei of HPMCs was found under extensive periods of PD. In immortal HPMCs, high glucose stimulated SRF overexpression in transformed fibroblastic HPMCs. SRF-siRNA preserved HPMC morphology, while transfection of SRF plasmid into HPMCs caused the opposite effects. PD with 4.25% glucose solution significantly induced SRF up-regulation and increased peritoneal thickness in rats. According to this, SRF translocation may be linked to HPMC EMT during the development of peritoneal fibrosis, suggesting that active SRF is necessary for EMT.

We also found that the inhibition of SRF signaling could prevent HPMCs from acquiring a spindle-like phenotype, reverse EMT, downregulate α-SMA and upregulate E-cadherin. To our knowledge, this report is the first to demonstrate a link between the translocation and activation of SRF in HPMCs and HG-induced EMT. In addition, SRF phosphorylation, which promotes SRF transcriptional activity, may have a role in EMT.

Snail is a zinc finger transcription factor expressed in cells and is the core EMT regulator that plays essential roles in fibrosis [31], [32]. The main mechanism by which Snail induces EMT is by downregulating E-cadherin [33]–[35]. In our study, we found cytoplasmic SRF translocated to the nucleus to induce Snail expression. We conclude that the subcellular localization of SRF and Snail may affect the changes in cellular morphology and behavior, which may be associated with altered signaling pathways, and the subsequent EMT-mediated fibrosis in the development of PD. We demonstrated high Snail expression in the peritoneal fibroblasts, confirming that such expression may play a substantial role in fibrosis progression. Evidence from electrophoretic mobility shift, chromatin immunoprecipitation and reporter assays further supported that SRF transcriptionally regulated Snail, a potent inducer of EMT, by directly binding to its promoter. Both SRE1 and SRE2, which can bind SRF, were predicted to be in the Snail promoter. However, SRE2 is the main binding site for SRF, and SRE1 plays a supporting role.

Moreover, there is still some limitation needed to show in our study. CCG-1423, a specific inhibitor of Rho pathway-mediated signaling and activation of SRF transcription, selectively inhibits DNA synthesis, proliferation and invasion of Rho-overexpressing cell lines. However, HG solutions could directly induce Rho kinases. Although a nuclear translocation of SRF in PD patients and a preservation of the peritoneal membrane in animals undergoing PD with HG solutions and receiving the inhibitor were shown in our study, any blockade of Rho kinases would reduce EMT and fibrosis, possibly irrespective of its specific SRF-dependent effects. So we added supplement data to demonstrate that Rho kinase inhibitor CCG-1423 has similar effects with SRF siRNA in vitro (Figure S2). It showed that inhibition of Rho kinase downstream SRF, has a similar effect with inhibition of Rho kinase per se on EMT and fibrosis, which are implied most of these are SRF-specific effect. In addition, so far we did not found any inhibitors to Rho kinase downstream, independent of SRF, so knockdown of SRF with SRF siRNA lentivirus in vivo is needed in future work.

In all, this study is the first extensive characterization of a signaling pathway that controls EMT in a non-tumor primary cell culture model and provides basic knowledge and clinical relevance. Our results from rats experiment reveal that, CCG-1423 could weaken the EMT happening of peritoneal mesothelial cells when stimulated by glucose contained in PD fluid, and protect peritoneal member function. These results could form the rationale for developing drugs that can counteract the progressive deterioration of the PM that occurs in CAPD patients. However, lots of work is needed to explore deeply molecular mechanism and to identify the safety and detail usage of drugs.

## Supporting Information

Figure S1 **The expression of SRF, E-cadherin and α-SMA were tested by Western blot after transfection by siRNA or SRF up-regulated plasmid in HPMCs which were stimulated by HG for 96 h.**
(TIF)

Figure S2 **CCG-1423 is stable in high glucose** ***in vitro*** **and** ***in vivo*** **.** (a) For in vivo study, we injected high glucose PDF after 2 hours of DMSO with CCG-1423 injection. The other progress is the same as before. The results by immunohistochemistry with PBS show that CCG-1423 still has a strong inhibition for peritoneal membrane proliferation even in high glucose solution. Magnification is 200×. (b) We demonstrated that Rho kinase inhibitor CCG-1423 has similar effects with SRF siRNA in vitro. Treated HPMCs with CCG-1423 after high glucose treatment (G1), before high glucose treatment (G2), as well as simultaneously treatment with high glucose (G3), CCG-1423 has a similar effect on SRF inhibition, and this result could be repeated with SRF siRNA (G4). Total incubation time of CCG-1423 or SRF-siRNA for each group was 72 hours.
(TIF)

Figure S3 **Inhibition of SRF by CCG-1423 ameliorates PD-induced PM fibrosis** ***in vivo*** **.** (a) Masson staining and expression of FN showed PD-induced PM fibrosis in Rats in vivo by immunohistochemistry. Magnification is 200×. (b) Westren blot staining of FN in HPMCs stimulated by HG reveals that PD fluid exposure induces the EMT and fibrosis process.
(TIF)

Table S1 **Characteristics of CAPD patients with ESRD in our study.**
(DOC)
