# Supplementary figures and images for: Serum Response Factor Accelerates the High Glucose-Induced Epithelial-to-Mesenchymal Transition (EMT) via Snail Signaling in Human Peritoneal Mesothelial Cells
Source: PLoS One. 2014 Oct 10;9(10):e108593. doi: 10.1371/journal.pone.0108593 (PMC4193747; doi:10.1371/journal.pone.0108593)

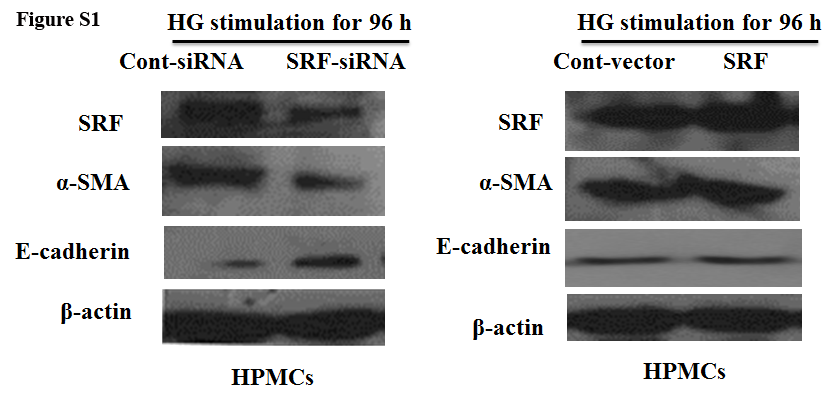

Supplement: Figure S1 — The expression of SRF, E-cadherin and α-SMA were tested by Western blot after transfection by siRNA or SRF up-regulated plasmid in HPMCs which were stimulated by HG for 96 h. (TIF) [file pone.0108593.s001.tif]

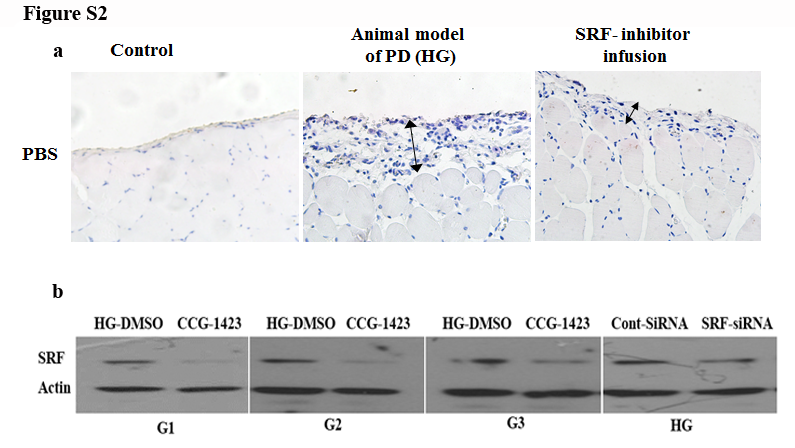

Supplement: Figure S2 — CCG-1423 is stable in high glucose in vitro and in vivo . (a) For in vivo study, we injected high glucose PDF after 2 hours of DMSO with CCG-1423 injection. The other progress is the same as before. The results by immunohistochemistry with PBS show that CCG-1423 still has a strong inhibition for peritoneal membrane proliferation even in high glucose solution. Magnification is 200×. (b) We demonstrated that Rho kinase inhibitor CCG-1423 has similar effects with SRF siRNA in vitro. Treated HPMCs with CCG-1423 after high glucose treatment (G1), before high glucose treatment (G2), as well as simultaneously treatment with high glucose (G3), CCG-1423 has a similar effect on SRF inhibition, and this result could be repeated with SRF siRNA (G4). Total incubation time of CCG-1423 or SRF-siRNA for each group was 72 hours. (TIF) [file pone.0108593.s002.tif]

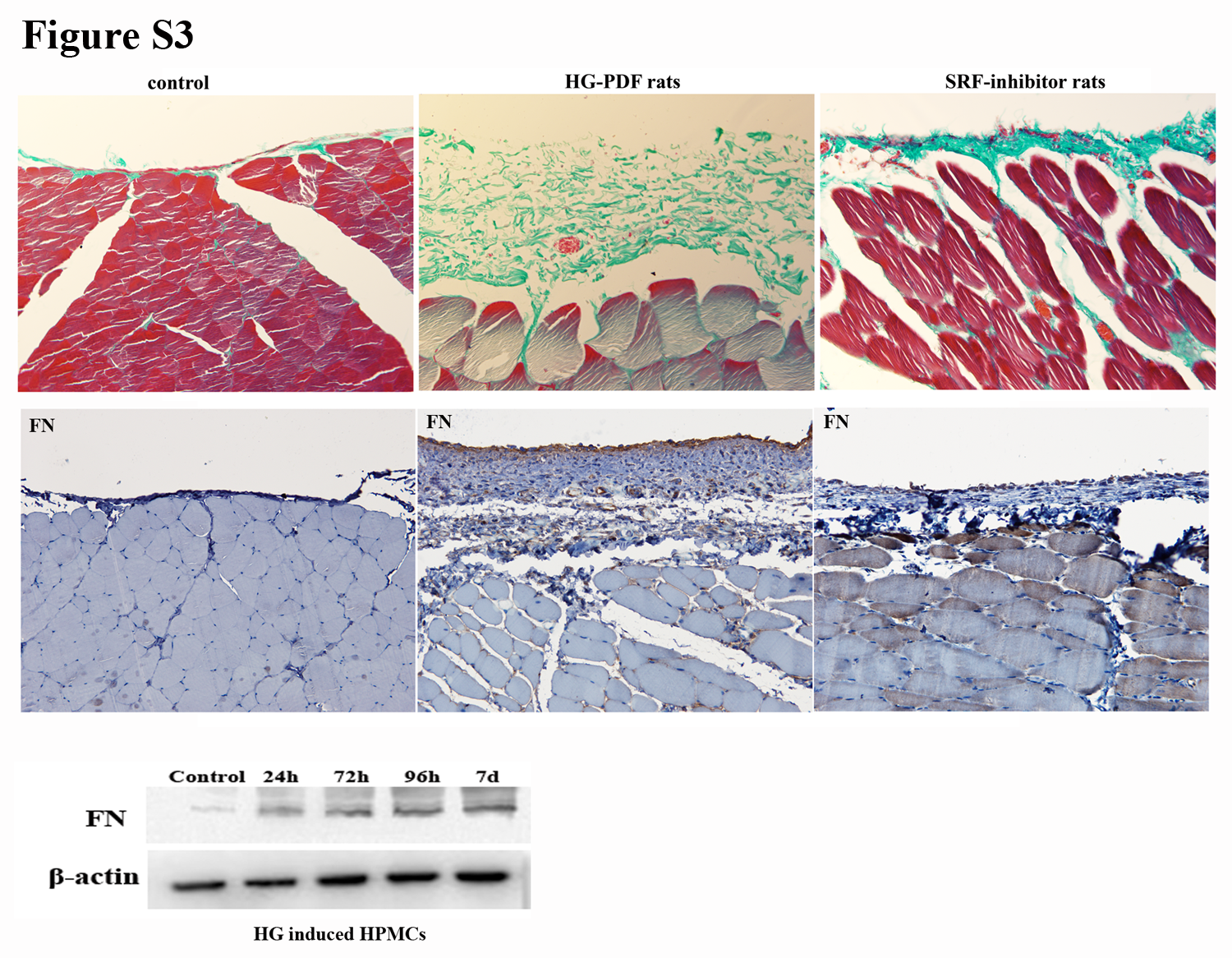

Supplement: Figure S3 — Inhibition of SRF by CCG-1423 ameliorates PD-induced PM fibrosis in vivo . (a) Masson staining and expression of FN showed PD-induced PM fibrosis in Rats in vivo by immunohistochemistry. Magnification is 200×. (b) Westren blot staining of FN in HPMCs stimulated by HG reveals that PD fluid exposure induces the EMT and fibrosis process. (TIF) [file pone.0108593.s003.tif]
