# Supplementary material for: Serum Response Factor Accelerates the High Glucose-Induced Epithelial-to-Mesenchymal Transition (EMT) via Snail Signaling in Human Peritoneal Mesothelial Cells
Source: PLoS One. 2014 Oct 10;9(10):e108593. doi: 10.1371/journal.pone.0108593 (PMC4193747; doi:10.1371/journal.pone.0108593)
Supplement: Table S1 — Characteristics of CAPD patients with ESRD in our study. (DOC) [file pone.0108593.s004.doc]

Table S1. Characteristics of CAPD patients with ESRD in our study

| Characteristic | NO.1 Group（n=17） | NO.2 Group  (n=10) | NO.3 Group  (n=15) | P |
| --- | --- | --- | --- | --- |
| CAPD times | 3-6 m | 6-24 m | ＞24 m |  |
| Age (yr) | 44.76±0.67 | 48.10±1.01 | 52.47±0.95 | 0.202 |
| Gender (M/F) | (11/6) | (10//0) | (9/6) | 0.369 |
| Primary cause of ESRD (%) |  |  |  | 0.542 |
| Diabetes | 2 | 0 | 3 |  |
| Hypertension | 2 | 0 | 1 |  |
| Glomerular Nephritis(GN) | 11 | 9 | 8 |  |
| other | 2 | 1 | 3 |  |
| Weight(kg) | 63.18±0.48 | 56.96±1.03 | 63.17±0.47 | 0.132 |
| BMI(kg/m2) | 22.31±0.13 | 20.27±0.33 | 23.03±0.18 | 0.043 |
| Hemoglobin (g/dl) | 103.29±1.37 | 92.30±2.73 | 96.67±1.42 | 0.484 |
| Serum creatinine (mmol/L) | 823.06±20.91 | 935.90±51.98 | 668.80±14.07 | 0.190 |
| Serum urea nitrogen (mmol/L) | 19.54±0.44 | 24.25±1.31 | 17.43±0.57 | 0.215 |
| Serum albumin (g/dl) | 38.26±0.35 | 36.63±0.35 | 36.91±0.21 | 0.594 |
